# Is microsatellite instability-high really a favorable prognostic factor for advanced colorectal cancer? A meta-analysis

**DOI:** 10.1186/s12957-019-1706-5

**Published:** 2019-10-21

**Authors:** Bingyan Wang, Fei Li, Xin Zhou, Yanpeng Ma, Wei Fu

**Affiliations:** Department of General Surgery, Peking University Third Hospital, Beijing, China

## Abstract

**Background:**

Stage II colorectal cancer with microsatellite instability-high (MSI-H) has been proven to have a better prognosis. However, in advanced stage, this trend remains controversial. This study aimed to explore the prognostic role of MSI-H in stage III and IV colorectal cancer (CRC) through meta-analysis.

**Methods:**

A comprehensive search was performed in PubMed, Cochrane Central Library, and Embase databases. All randomized clinical trials and non-randomized studies were included based on inclusion and exclusion criteria and on survival after a radical operation with or without chemotherapy. The adjusted log hazard ratios (HRs) were used to estimate the prognostic value between MSI-H and microsatellite-stable CRCs. The random-effects model was used to estimate the pooled effect size.

**Results:**

Thirty-six studies were included. Randomized controlled trials (RCT) and non-RCT were analyzed separately. For stage III CRCs, pooled HR for overall survival (OS) was 0.96 (95% confidence interval [CI] 0.75–.123) in the RCT subgroup and 0.89 (95% CI 0.62–1.28) in the non-RCT subgroup. For disease-free survival (DFS), the HR for the RCT group was 0.83 (95% CI 0.65–1.07), similar to the non-RCT subgroup (0.83, 95% CI 0.65–1.07). Disease-specific survival (DSS) was also calculated, which had an HR of 1.07 (95% CI 0.68–1.69) in the non-RCT subgroup. All these results showed that MSI-H has no beneficial effects in stage III CRC. For stage IV CRC, the HR for OS in the RCT subgroup was 1.23 (95% CI 0.92–1.64) but only two RCTs were included. For non-RCT study, the combined HR for OS and DFS was 1.10 (95% CI 0.77–1.51) and 0.72 (95% CI 0.53–0.98), respectively, suggesting the beneficial effect for DFS and non-beneficial effect for OS.

**Conclusion:**

For stage III CRC, MSI-H had no prognostic effect for OS, DFS, and DSS. For stage IV CRC, DFS showed a beneficial result, whereas OS did not; however, the included studies were limited and needed further exploration.

## Background

Colorectal cancer (CRC) is the third most common cancer worldwide [1]. Due to the heterogeneity of the disease, various factors are proven to be associated with the prognosis in CRC patients. The Cancer Genome Atlas (TCGA) program classified CRC into two large groups: chromosomal instability (CIN) and microsatellite instability (MSI) [2]. MSI is the alteration of the size of nucleotide repeat sequence named microsatellites, which is caused by the loss-of-function of mismatched repair (MMR) gene; leading to the inability to repair DNA mismatches and accounted for approximately 15 to 20% of CRC patients.

The National Comprehensive Cancer Network (NCCN) guidelines [3] stated that stage II MSI-H patients have a better prognosis and do not benefit from fluorouracil (5-FU) adjuvant therapy [4]. Unlike microsatellite-stable (MSS) CRCs, MSI-H not only had a much more active immune microenvironment with greater tumor-infiltrating lymphocytes (TIL), but also showed cancer-specific upregulation of inhibitory checkpoints including programmed cell death protein 1 (PD-1) and CTLA4 [5]. Therefore, unlike MSS CRCs, MSI-H CRCs showed a much better response to checkpoint immunotherapy. It is interesting to note that there are lots of controversies about whether microsatellite instability-high (MSI-H) is a good prognostic factor in stage III and stage IV CRC patients. Some studies proved that MSI-H is still a beneficial factor with better oncological survival [6, 7]. However, several researches came to opposite conclusions, indicating MSI-H as an adverse factor for both overall survival (OS) and cancer-related survival [8].

MSI status can be confirmed by polymerase chain reaction (PCR) with the results of MSI-H or MSS. However, the PCR method is expensive and complicated, while immunohistochemistry(IHC) method is cheap, convenient, and widely used [9]. IHC can prove whether there is a mismatch repair deficiency (dMMR) that indicates a similar situation as MSI-H, and previous research has proved that these two methods have excellent agreement [10].

In order to further explore the prognostic value of MSI-H in stage III and stage IV colorectal cancer patients, a comprehensive meta-analysis was performed.

## Materials and methods

Two authors searched the PubMed electronic database, Cochrane Central Library database, and Embase for available articles that were published before July 2018. Search terms covered four aspects considering the variants of the following keywords, which included “colorectal cancer,” “microsatellite instability,” “advanced stage,” and “survival.” The PubMed search terms are listed as follows: (((((((Colonic Neoplasms) OR Colorectal Neoplasms) OR Colorectal cancer) OR colon cancer)) AND (((((Microsatellite Instability) OR Microsatellite Repeats) OR MSI) OR Mismatch repair) OR dMMR)) AND (((((((Neoplasm Metastasis) OR lymphatic metastasis) OR late stage) OR stage III OR stage IV OR advanced stage) OR metastasis)) AND ((((((prognosis) OR mortality) OR survival) OR OS) OR DFS) OR outcome). The search strategy was modified accordingly for the Cochrane Central Library database and Embase.

Inclusion criteria were listed as follows:

1. Original articles, with retrievable survival data in full text or abstract, that compare the clinical outcome between MSI-H and MSS in stage III or stage IV CRC.
2. From the abstract or full text, hazard ratio (HR) of OS, disease-free survival (DFS), or other survival rates between MSI-H and MSS groups, can be acquired, or calculated.

Also, research that matched any of the criteria below were excluded to prevent bias.

1. Patients who received immunotherapy such as anti-PD-1 or anti-programmed death-ligand 1 (PD-L1) treatment.
2. When the number of patients in the MSI-H group was less than 9.
3. Research that included other factors (such as BRAF status) that mixed with microsatellite status and could not calculate the HR separately.

The search and analysis procedures were performed by two authors separately. If multiple researches were used to investigate the patients in the same clinical trial or medical institution, the latest or largest one will be included in order to prevent overlapping. We also excluded letters, review articles, and case reports. If the two authors had a disagreement, a third reviewer made the decision.

The included studies comprised of both RCT and non-RCT studies; therefore, both Cochrane and Newcastle–Ottawa scale were used to assess the methodological quality

### Statistical analysis and data synthesis

Considering the different clinical survivals in stage III and IV CRC, the analysis for OS, DFS, and disease-specific survival (DSS) were performed separately according to the different stages.

The adjusted log hazard ratios (HRs) were used to estimate the prognostic value between MSI-H and MSS CRCs. The HRs were extracted from the Cox proportional hazards regression model provided in the included articles. For studies that failed to provide the HR value between MSI-H and MSS groups but provided the Kaplan-Meier survival curves, Engauge Digitizer (Version 4.1) was used to extract the survival information from the curve while HR was calculated by the method provided by Tierney et al. [11]

Meta-analysis was performed between MSI-H and MSS patients to explore the relationship between microsatellite status and clinical prognosis, using Stata version 14.0 (Stata, College Station, TX).

Heterogeneity was quantified using the *I*^2^ statistic. The random-effects model was conducted to estimate the pooled effect size of OS, DFS, and DSS. Funnel plots were performed in every analysis to examine publication bias. Sensitivity analysis was also performed in every subgroup analysis. Meta-regression analysis was performed to control for heterogeneity. The DFS is usually defined based on the “study entry till documented progression or death from any cause,” while relapse-free survival (RFS) and progression-free survival (PFS) showed similar endpoints. Therefore, these data were analyzed together. For the same season, we combined the DSS and cancer-specific survival (CSS).

The RCT studies were assessed according to the Cochrane protocol. The non-RCT studies were assessed using the Newcastle–Ottawa scale which considers participant selection, comparability, and outcome with a full score of 9. A score higher than 6 was considered to be of good quality for the individual study.

## Results

**Fig 1:**
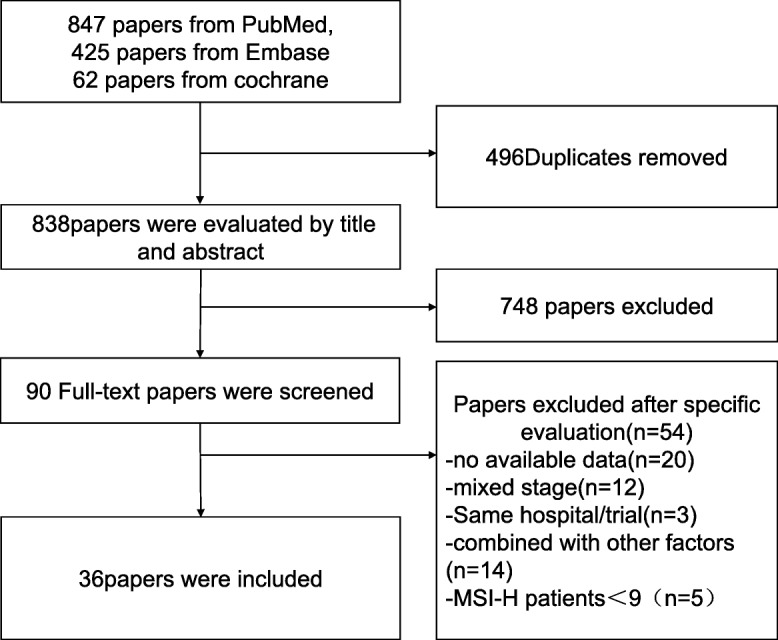
Flow diagram of researches screening

Overall, 847, 425, and 62 studies were retrieved from PubMed electronic database, Embase, and Cochrane Library, respectively. After the removal of duplicate articles, a total of 838 papers that matched the inclusion criteria were found. In total, 748 papers were excluded following the reading of the title and abstract. The full text of the remaining 90 articles was carefully read by two authors. Several researches were excluded due to the inability to acquire the specific survival data. Finally, 36 articles, that provided specific survival information, were included in this meta-analysis (Fig. 1).

### Characteristics of the studies

**Fig 2:**
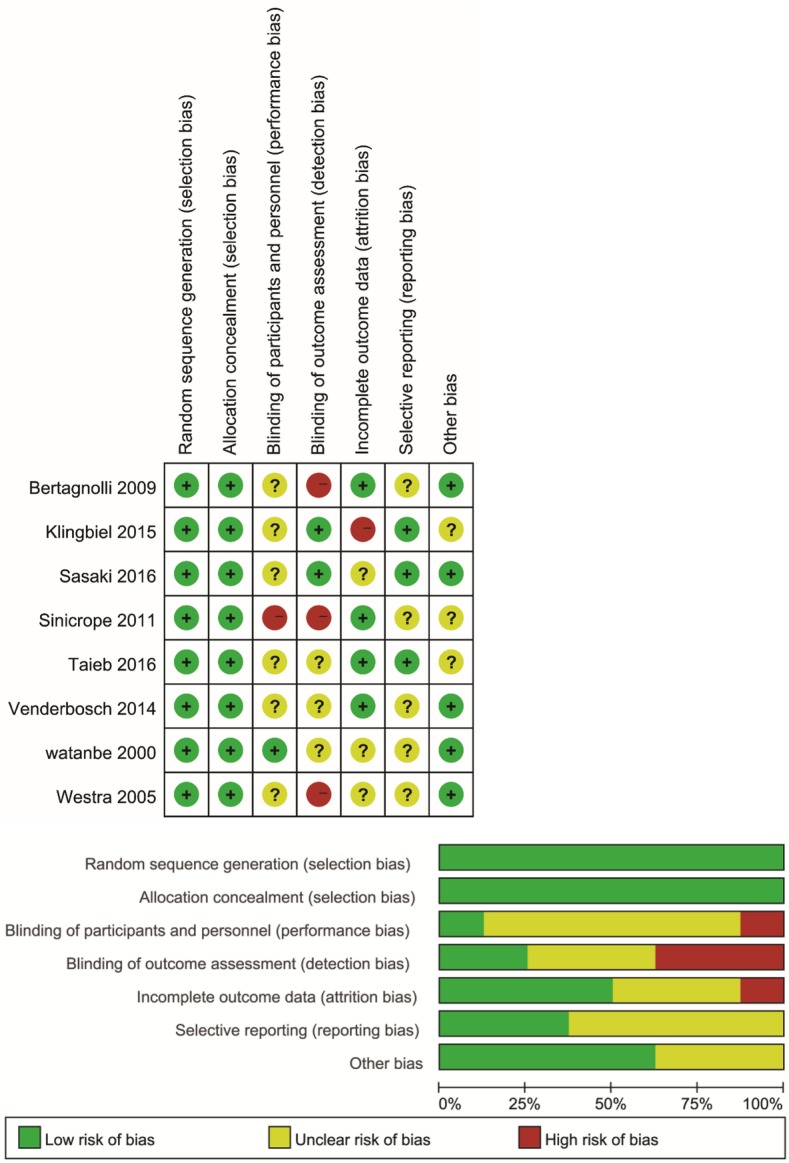
Cochrane risk of bias analysis for included randomized controlled trial

The characteristics of the studies are summarized in Table 1. For stage III CRC, seven [6, 8, 10, 33, 34, 36, 39] RCTs had survival information for both OS and DFS; and an extra article [40] was available for DFS analysis. There were 13 [7, 15, 16, 18, 19, 21, 23, 25, 29, 32, 41, 43, 44] non-RCT studies that provided information on OS HR or available data while 11 [15, 18, 19, 21, 23–25, 32, 37, 42, 43] were found for DFS analysis. Five researches [13, 20, 28, 29, 37] also provided DSS/CSS information. For stage IV, only two RCTs [30, 31] for OS were available, with eight non-RCTs [12, 14, 17, 22, 25–27, 38]. No available RCT data for DFS analysis and five non-RCT [22, 25–27, 30] articles were included. Five studies [13, 20, 28, 29, 37] have CSS/DSS information and were analyzed together. The MSI could be measured by both PCR and IHC, different researches performed different methods as shown in Table 1. Cochrane risk of bias and Newcastle–Ottawa scale results are shown in Fig. 2 and Table 2; both of them showed no obvious risk of bias.

**Table 1:** Characteristics of the included studies

Author	Country	Year	Study design	Stage	Total no.	MSI-H	MSS	MSI determination	Survival information	Chemotherapy
Alex, A.K [12]	Brazil	2017	Non-RCT	IV	126	42	84	IHC + PCR	OS	Oxaliplatin-based
Bertagnolli [10]	USA	2009	RCT	III	702	96	606	IHC + PCR	OS, DFS	FU/LV/IFL
Chouhan [13]	Australia	2018	Non-RCT	III	686	95	591	IHC + PCR	CSS	NA
des Guetz [14]	France	2007	Non-RCT	IV	40	9	31	PCR	OS	FOLFOX
Drucker [15]	Canada	2013	Non-RCT	III	159	18	141	IHC + PCR	OS, DFS	FOLFOX/capecitabine
Elsaleh [16]	Australia	2001	Non-RCT	III	732	63	669	PCR	OS	5-FU/levamisole
Fujiyoshi [17]	Japan	2017	Non-RCT	IV	401	15	386	PCR	OS	NA
Guidoboni [7]	Italy	2001	Non-RCT	III	54	20	34	PCR	OS	5-FU
Hemminki [18]	Finland	2000	Non-RCT	III	95	11	84	PCR	OS, DFS	5-FU-based
Jover [19]	Spain	2006	Non-RCT	III	209	18	191	IHC + PCR	OS, DFS	5-FU-based
Jung [20]	Korea	2016	Non-RCT	III	60	19	41	PCR	CSS	NA
Kim, C.G [21].	Korea	2016	Non-RCT	III	2940	261	2679	PCR	OS, DFS	5-FU/LV/FOLFOX
Kim, J.E [22].	Korea	2011	Non-RCT	IV	197	23	174	IHC + PCR	OS, DFS	FOLFIRI/XELIRI
Kim, J.E [23].	Korea	2017	Non-RCT	III and IV	795	73	722	PCR	OS, DFS	FOLFOX
Kim, S.H [24].	Korea	2013	Non-RCT	III	394	26	368	PCR	DFS	FOLFOX
Klingbiel, D [6].	Switzerland	2015	RCT	III	859	104	755	PCR	OS, DFS	5-FU/LV/FOLFIRI
Li, P [25].	China	2017	Non-RCT	III and IV	599	54	545	IHC	OS, DFS	FOLFOX/XELOX
Liu [26]	China	2018	Non-RCT	IV	461	30	431	IHC + PCR	OS, DFS	NA
Ma, J [27].	China	2015	Non-RCT	IV	184	34	150	IHC	OS, PFS	FOLFIRI/irinotecan
Malesci, A [28].	Italy	2007	Non-RCT	III	264	27	237	PCR	DSS	5-FU
Mohan, H.M [29].	Ireland	2016	Non-RCT	III	320	32	288	IHC + PCR	OS, DSS	NA
Nopel-Dunnebacke [30]	Germany	2014	RCT	IV	204	14	190	IHC + PCR	OS, PFS	CAPOX/FUFOX
Nordholm-Carstensen [31]	Denmark	2015	RCT	IV	935	75	860	IHC	OS	NA
Oh, S.Y [32].	Korea	2013	Non-RCT	III	127	16	111	PCR	OS, DFS	FOLFOX
Sasaki, Y [33].	Japan	2016	RCT	III	304	23	281	IHC	OS, RFS	UFT
Sinicrope, F. A [34]	USA	2011	RCT	III	1363	180	1183	IHC + PCR	OS, DFS	5-FU-based
Sinicrope, F.A [35].	USA	2013	RCT	III	2580	314	2266	IHC + PCR	DFS	FOLFOX-based
Taieb, J [36].	France	2016	RCT	III	1791	177	1614	IHC + PCR	OS, DFS	FOLFOX ± cetuximab
Tan, W. J [37].	Singapore	2018	Non-RCT	III	299	27	272	IHC	DSS, RFS	5FU/capecitabine ± oxaliplatin
Tran, B [38].	Australia	2011	Non-RCT	IV	350	40	310	IHC + PCR	OS	NA
Venderbosch, S [8].	Netherlands	2014	RCT	III	3063	153	2910	IHC	OS, PFS	NA
Watanbe [39]	USA	2000	Non-RCT	III	229	73	156	PCR	OS, DFS	5-FU–based
Westra, J. L. [40]	UK	2005	RCT	III	273	229	44	PCR	DFS	5-FU–based
Wright, C.M [41].	Australia	2000	Non-RCT	III	238	21	217	PCR	OS	NA
Zaanan, A [42].	France	2010	Non-RCT	III	233	32	201	IHC + PCR	DFS	FOLFOX
Zaanan, A [43].	France	2011	Non-RCT	III	303	34	269	IHC + PCR	OS, DFS	FOLFOX

*MSI-H* microsatellite instability-high, *MSS* microsatellite stable, *RCT* randomized controlled trial, *IHC* immunohistochemistry, *PCR* polymerase chain reaction, *OS* overall survival, *DFS* disease-free survival, *DSS* disease-specific survival, *CSS* cancer-specific survival, *RFS* recurrence-free survival, *FU* fluorouracil, *LV* leucovorin, *IFL* irinotecan + fluorouracil + leucovorin, *5-FU* 5-fluorouracil, *FOLFOX* 5-fluorouracil + leucovorin + oxaliplatin, *FOLFIRI* 5-fluorouracil + irinotecan + leucovorin, *XELOX* xeloda + oxaliplatin, *CAPOX* capecitabine + oxaliplatin, *FUFOX* fluorouracil + fludarabine + oxaliplatin, *UFT* tegafur

**Table 2:** Newcastle–Ottawa scale for included non-RCT studies

Author	Year	Study design	Selection				Comparability		Outcome			total
			1	2	3	4	1	2	1	2	3	
Alex, A.K [12]	2017	Non-RCT	1	1	1	1	1	1	1	1	1	9
Chouhan [13]	2018	Non-RCT	1	1	1	1	1	0	1	1	1	8
des Guetz, G [14]	2007	Non-RCT	1	1	1	1	1	1	0	1	1	8
Drucker, A [15]	2013	Non-RCT	1	1	1	1	1	0	1	1	0	7
Elsaleh, H [16]	2001	Non-RCT	1	1	1	1	1	1	0	1	1	8
Fujiyoshi, K [17]	2017	Non-RCT	1	1	1	1	1	1	1	0	1	8
Guidoboni, M [7].	2001	Non-RCT	1	1	1	1	1	0	1	1	1	8
Hemmink i [18]	2000	Non-RCT	1	1	1	1	1	1	1	1	1	9
Jover [19]	2006	Non-RCT	1	1	1	1	1	0	1	0	1	7
Jung, S.H [20].	2016	Non-RCT	1	1	1	1	1	1	1	1	1	9
Kim, C. G [21].	2016	Non-RCT	1	1	1	1	1	0	1	1	1	8
Kim, J.E [23].	2017	Non-RCT	1	1	1	1	1	1	0	1	1	8
Kim, J.E [22].	2011	Non-RCT	1	1	1	1	1	1	1	1	1	9
Kim, S.H [24].	2013	Non-RCT	1	1	1	1	1	0	1	0	1	7
Lanza, G [44].	2006	Non-RCT	1	1	1	1	1	1	0	1	0	7
Li, P [25].	2017	Non-RCT	1	1	1	1	1	1	1	1	1	9
Liu [26]	2018	Non-RCT	1	1	1	1	1	1	1	1	1	9
Ma, J [27].	2015	Non-RCT	1	1	1	1	1	1	1	1	1	9
Malesci, A [28].	2007	Non-RCT	1	1	1	1	1	0	1	1	0	7
Mohan, H.M [29].	2016	Non-RCT	1	1	1	1	1	1	1	1	1	9
Oh, S.Y [32].	2013	Non-RCT	1	1	1	1	1	0	1	0	1	7
Tan, W. J [37].	2017	Non-RCT	1	1	1	1	1	1	0	1	1	8
Tran, B [38].	2011	Non-RCT	1	1	1	1	1	1	1	1	1	9
Wright, C. M [41].	2000	Non-RCT	1	1	1	1	1	0	1	1	1	8
Zaanan, A [43].	2011	Non-RCT	1	1	1	1	1	1	0	0	0	6
Zaanan, A [42].	2010	Non-RCT	1	1	1	1	1	1	0	1	1	8

### Data analysis

**Fig 3:**
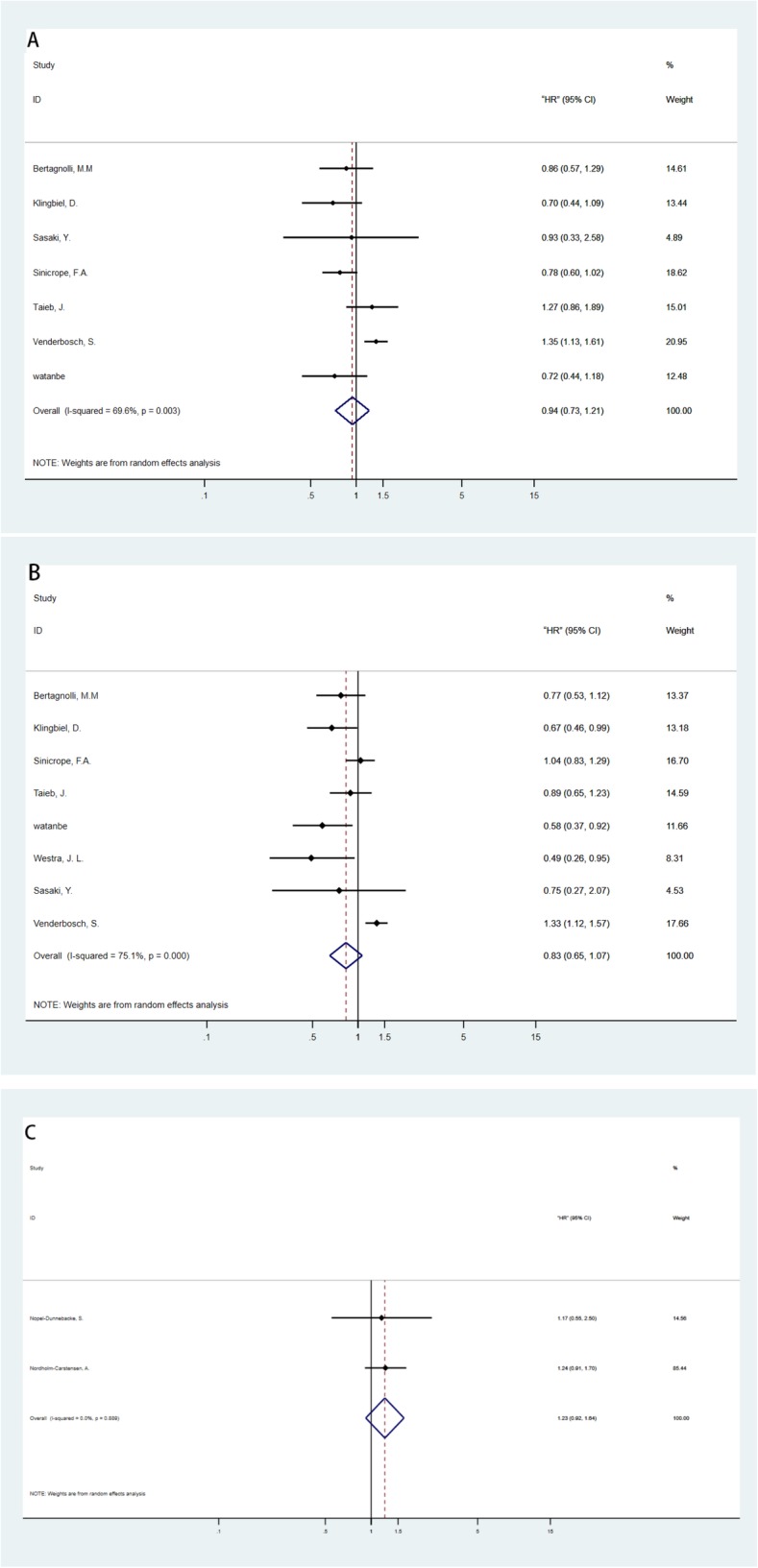
Meta-analysis of HRs between microsatellite instability-high (MSI-H) and microsatellite-stable (MSS) CRC patients in randomized controlled trials (RCTs). **a** Overall survival (OS) for stage III. **b** Disease-free survival (DFS) for stage III. **c** OS for stage IV

**Fig 4:**
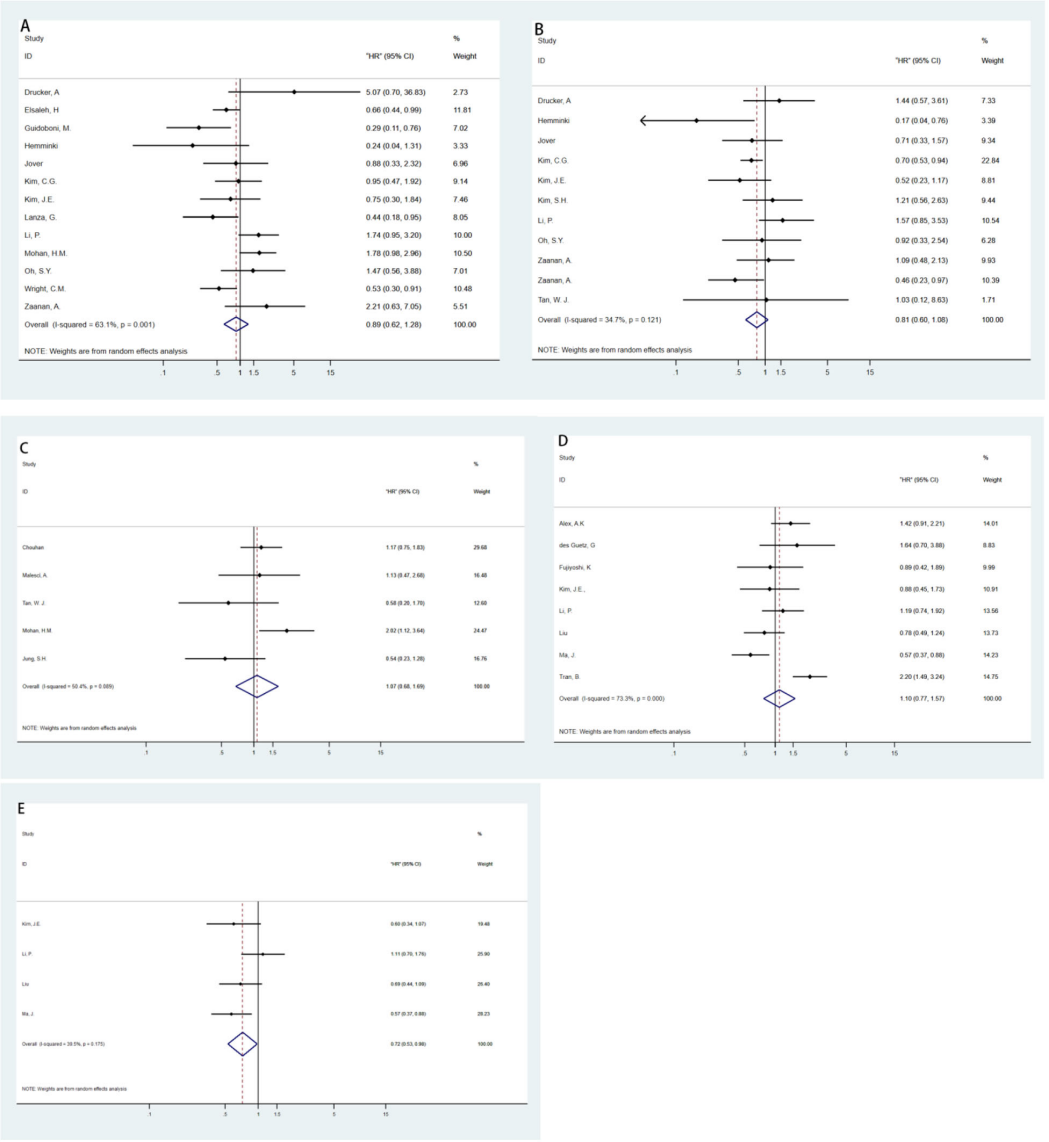
Meta-analysis of HRs between microsatellite instability-high (MSI-H) and microsatellite-stable (MSS) CRC patients in non-randomized controlled trials (non-RCTs). **a**–**c** Overall survival (OS); disease-free survival (DFS); disease-specific survival (DSS) for stage III. **d**–**e** OS and DFS for stage IV

Meta-analyses were performed for every subgroup according to stage, study method, and survival information. Figure 3 shows the results of the RCTs while Fig. 4 presents the results of the non-RCTs.

### Relationship between MSI and survival

The RCT and non-RCT studies were analyzed separately both for stage III and stage IV groups. The analysis used OS, DFS, and DSS as the judging point. Since DFS, PFS, and RFS have very similar endpoints, their data were analyzed together. Also, DSS and CSS were analyzed together.

For stage III CRC, the calculated HR value of OS was 0.94 (95% CI 0.73–1.21) in RCT subgroup and 0.89 (95% CI 0.62–1.28) in non-RCT subgroup. For DFS, the RCT group showed HR of 0.83 (95% CI 0.65–1.07) similar to the non-RCT subgroup, 0.83 (95% CI 0.65–1.07). DSS was also calculated as 1.07 (95% CI 0.68–1.69) in the non-RCT subgroup. All these results showed that MSI-H had no beneficial effect in stage III CRC.

For stage IV CRC, the HR for OS in the RCT subgroup was 1.23 (95% CI 0.92–1.64) but only two RCTs were included. For non-RCT study, the combined HR for OS and DFS was 1.10 (95% CI 0.77–1.51) and 0.72 (95% CI 0.53–0.98), respectively. The pooled HR suggested a non-beneficial effect for OS. However, for DFS, the pooled results suggested a slight beneficial effect.

### Sensitivity analysis

**Fig 5:**
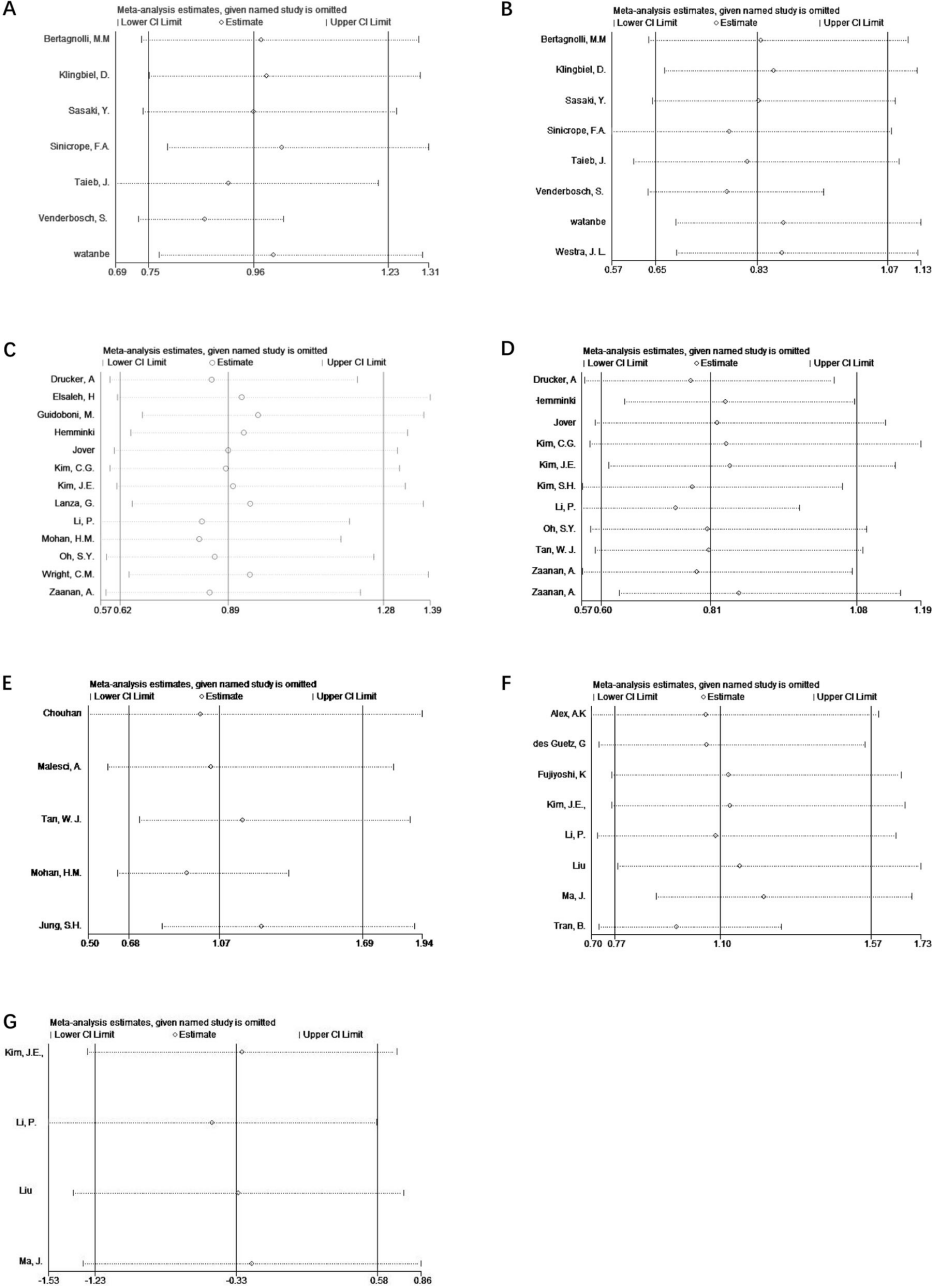
Sensitivity analysis for included researches. **a** Overall survival (OS) for stage III RCT studies. **b** Disease-free survival (DFS) for stage III RCT studies. **c** OS for stage III retrospective studies. **d** DFS for stage III retrospective studies. **e** Disease-specific survival (DSS) for stage III retrospective studies. **f** OS for stage IV retrospective studies. **g** DFS for stage IV retrospective studies

Sensitivity analysis was performed for all subgroups, each study was excluded to draw a new result that is shown in Fig. 5; and no obvious bias was detected in the subgroup analysis.

**Fig 6:**
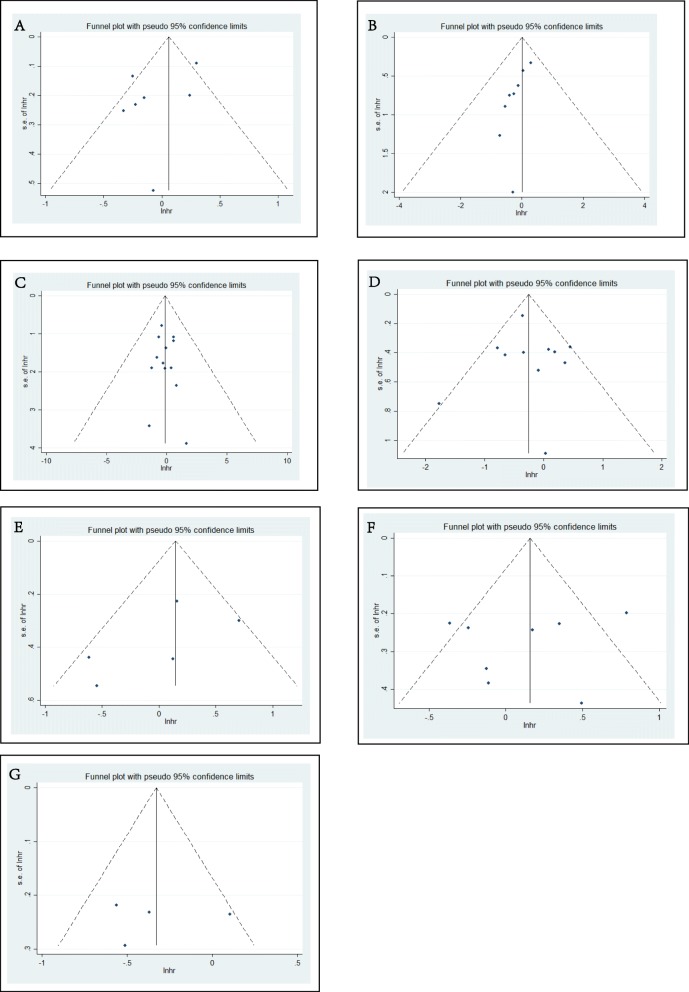
Publication bias detected by funnel plots. The funnel plots showed the HR associated with MSI in included studies of different subgroups. **a** Overall survival (OS) for stage III RCT studies. **b** Disease-free survival (DFS) for stage III RCT studies. **c** OS for stage III retrospective studies. **d** DFS for stage III retrospective studies. **e** Disease-specific survival (DSS) for stage III retrospective studies. **f** OS for stage IV retrospective studies. **g** DFS for stage IV retrospective studies

Publication bias was detected in all subgroups. The results are shown in the funnel plots (Fig. 6); and no obvious publication bias was detected.

Since the included studies in stage IV were limited, meta-regression analysis was performed for stage III researches. Study design and year of publication were taken into consideration. The adjusted *I*^2^ was 36.55% for OS and 51.26% for DFS, suggesting study design and year of publication to be influencing factors.

## Discussion

For stage III CRC, neither RCTs nor retrospective studies reached a convincing conclusion. Most included RCTs had insignificant results, except Venderbosch’s [8] research, which showed that dMMR conferred an inferior prognosis on both DFS and OS based on a series of cohort studies including 3063 patients. On the contrary, Klingbiel et al. [6] and Westra [40] studies showed MSI-H to be a good factor for DFS. The synthetized analysis turned out to be inconclusive. For non-RCTs, several researches revealed statistically significant results; however, the pooled result failed to draw a positive result.

For stage IV CRC, there were only two RCTs available [30, 31] on OS, both of which showed no positive results. Retrospective studies also showed no conclusive results of the predictive effect on OS. This indicates MSI-H/dMMR not to be predictive factor of a better prognosis. For DFS, the only RCT [30] showed no survival difference (*p* = 0.47) between MSI-H and MSS group. Non-RCT researches revealed a significant beneficial result, but the included researches were limited and the number of MSI-H patients was few, making the result less convincing. Another point was that the definition of stage IV was too wide and the survival rate about whether a patient can achieve “no evidence of disease” (NED) differed greatly. It was discovered that in most studies, NED patients were not separated from non-surgical patients during analysis, several studies only included unresectable patients, making the results of stage IV less convincing. Therefore, whether MSI-H is beneficial for stage IV DFS needs further exploration with large scale RCTs.

To our knowledge, this meta-analysis is the first to summarize the prognostic effect of MSI-H CRC in an advanced stage. The result showed that MSI-H may not be a good prognostic factor for stage III or stage IV CRC patients.

Although MSI-H CRC accounted for only about 15% of all CRC patients, this special molecular subtype has a distinctly different pathological manifestation including poor differentiation, accumulation of lymphocytes, and intertumoral heterogeneity. The NCCN guideline indicates that stage II MSI-H patients may have a good prognosis and do not benefit from 5-FU adjuvant therapy [3]. For stage III and IV CRC, there are disagreements on whether MSI-H is a good prognostic factor.

Recent studies [45, 46] proved that MSI-CRCs were sensitive to immune checkpoint blockade with anti-PD1 and PD-L1 antibodies. dMMR patients have much higher somatic mutations and prominent lymphocyte infiltrates. Previous studies showed that MSI-H CRCs exhibit a strong association with tumor-infiltrating lymphocytes and the immune reaction is strongly relevant to survival [47, 48]. This may explain why early-stage MSI-H CRCs manifest a better clinical prognosis. Furthermore, this paradoxical phenomenon can be explained by a lot of studies focused on the immune checkpoint. While MSI-H CRCs have more tumor-infiltrating lymphocytes, scientists also found MSI-H tumor microenvironment strongly expressed several checkpoint ligands including PD-1 and CTLA-4. However, the active immune microenvironment is counterbalanced by immune inhibitory signals that resist tumor elimination [5]. This may explain why stage III and IV MSI-H CRC did not manifest a better survival, it might be because the immune system has completely lost the fight against tumor cells who overexpressed PD-1 and CTLA-4, and then metastasis began [49–51]. One important thing to notice is that MSI-H has a strong relevance in the upregulation of immune checkpoint, making checkpoint inhibitor a promising treatment method [52].

As mentioned above, MSI CRC accounted for about 15% of all CRC patients. However, in this meta-analysis, the percentage of MSI-H patients was 11% and 9.4% in stage III and stage IV subgroup, respectively. The lower percentage may suggest that MSI-H/dMMR CRC have a reduced potential of metastasis [28, 53], but the underlying mechanism is yet to be clarified. One plausible explanation could be the stronger immunoreaction of MSI-H cancer [54].

Several aspects of this meta-analysis warrant further discussions. For advanced-stage CRC, chemotherapy was commonly recommended, but the chemotherapy regimen has altered a lot in recent decades; this may cause different effect on MSI-H and MSS patients. Therefore, we also analyzed the prognostic effect of different chemotherapy on these subgroups of patients; but there were no significant conclusions.

There are several limitations to this meta-analysis. First, the majority included researches that were non-RCTs due to the limited number of RCTs. Secondly, there was heterogeneity between the included studies and exaggeration may still exist even with the random-effects model. On the contrary, sensitivity analysis did not show obvious change in the pooled results, suggesting an acceptable result.

Thirdly, several researches did not provide HR, and relative survival data were extracted for the article in order to calculate the approximate HR. Although mathematically practical, this may cause a slight calculation error. Lastly, the researches focusing on stage IV CRC is very limited; therefore, this may not reach a very convincing result.

In conclusion, in contrast to stage II, MSI-H CRCs showed no good prognostic effect for OS, DFS, and DSS in stage III as well as OS for stage IV CRCs patients.

## Data Availability

All data were extracted from published articles, and the datasets supporting the conclusions of this article are included within the article and its additional files.

## Abbreviations

5-FU: 5-fluorouracil
CAPOX: Capecitabine + oxaliplatin
CSS: Cancer-specific survival
DFS: Disease-free survival
DSS: Disease-specific survival
FOLFIRI: 5-fluorouracil + irinotecan + leucovorin
FOLFOX: 5-fluorouracil + leucovorin + oxaliplatin
FU: Fluorouracil
FUFOX: Fluorouracil + fludarabine + oxaliplatin
IFL: Irinotecan + fluorouracil + leucovorin
IHC: Immunohistochemistry
LV: Leucovorin
MSI-H: Microsatellite instability-high
MSS: Microsatellite stable
OS: Overall survival
PCR: Polymerase chain reaction
RCT: Randomized controlled trial
RFS: Recurrence-free survival
UFT: Tegafur
XELOX: Xeloda + oxaliplatin
